# Culturing Potential: advances in ex vivo cell culture systems for haematopoietic cell-based regenerative therapies

**DOI:** 10.1016/j.reth.2025.07.001

**Published:** 2025-07-17

**Authors:** Ayano Sugiyama-Finnis, Satoshi Yamazaki

**Affiliations:** Division of Cell Regulation, Center for Experimental Medicine and Systems Biology, The Institute of Medical Science, The University of Tokyo, Minato-ku, Tokyo, 108-8639, Japan

## Abstract

Stem-cell derived therapies are an essential pillar in the field of regenerative medicine, utilising stem cell self-renewal and multipotent or pluripotent differentiation capabilities to give rise to functional, specialised cells to repair and restore tissue function. Haematopoietic cell therapies have been pivotal to the development of the regenerative medicine field and continue to hold significant promise enabled by recent technical innovation in cell culture approaches that have expanded their therapeutic potential. The development of novel cell culture protocols that allow for the standardised ex vivo expansion of haematopoietic stem cells (HSCs) has facilitated the exploration of umbilical cord blood allogeneic HSC transplantation. Directed differentiation protocols of HSCs, embryonic stem cells and induced pluripotent stem cells, to selectively produce a desired haematopoietic cell type in a donor-independent manner, has broadened the scope for haematopoietic cell-based regenerative therapy. Furthermore, the integration of genome modification or gene editing with these protocols have allowed for corrective autologous HSC transplantation as well as the ability to confer haematopoietic cells with enhanced or novel therapeutic functions. Despite this, realising large-scale clinical translation remains challenging. Current efforts aim to move towards chemically defined culture systems, improving the efficiency and reproducibility of lineage-specific differentiation with an emphasis on compatibility with genome modification and gene-editing protocols for the scalable production of high-quality, efficacious and safe cellular therapies. In this review, we summarise the key milestones and technical advancements in the field in addition to the outstanding questions to be addressed.

## 1 Introduction

Regenerative medicine is an interdisciplinary field encompassing stem cell biology, genetic engineering and biomaterial development with the aim to replace or repair diseased tissue to restore function [1]. Stem-cell derived therapies, an essential pillar of regenerative medicine, utilise stem-cell self-renewal and multipotent or pluripotent differentiation capabilities as a basis to promote regeneration in the treatment of pathology such as cancer or cardiovascular disease [2]. These therapies can directly give rise to the differentiated and specialised cellular components of tissues to facilitate regeneration through stem cell transplantation and transfer of ex vivo differentiated cells or indirectly through promoting physiological regenerative mechanisms such as endogenous stem cell activation [3].

Historically, haematopoietic cells have been instrumental in the development of regenerative medicine [2]. The first example of a regenerative therapy, an allogeneic haematopoietic stem cell transplantation (HSCT) in the treatment of acute myeloid leukaemia and acute lymphoblastic leukaemia [4], followed the initial demonstration of murine haematopoietic reconstitution following irradiation by a syngeneic bone marrow graft [5]. The role of haematopoietic cells in regenerative therapies has continued to evolve, utilising multipotent stem cells such as haematopoietic stem cells (HSCs) or human pluripotent stem cells (hPSCs) such as embryonic stem cells (ESCs) and induced pluripotent stem cells (iPSCs). These therapies may aim to regenerate either the entire haematopoietic system or specific populations of differentiated haematopoietic cell with physiological, enhanced or novel functions.

Continued research into haematopoietic cells for the development and scalability of regenerative therapies remain pertinent and have been central to advancing the field. Of current interest is the potential for regenerated haematopoietic cells to target pathology beyond immediate haematological disease, for example through crosstalk with the wider physiology, such as immunotherapy for cancer [6] or autoimmune disease [7]. In addition, the established nature of ex vivo cell culture systems [8] have encouraged the application of advanced cell culture [9] and genome modifying technologies [10] to haematopoietic cells while accepted HSCT and haematopoietic cell transfer protocols allow for reproducible functional assays [8], contributing to the development of novel regenerative therapies.

Despite this progress, limitations to the large-scale clinical translation of these therapies remain. This includes reproducibility concerns arising from ex vivo culture using serum or feeder cells during manufacture [11], incomplete or artefactual cell differentiation posing safety or efficacy concerns such as immunogenicity, tumorigenesis and impaired or incomplete cellular function [12]. In attempts to address these issues, various innovative cell culture systems have been developed. Examples have included the supplementation of culture medium with epigenetic inhibitors to improve HSC expansion or maintenance of function [13] and the development of chemically defined, feeder-free culture systems as the gold standard for ex vivo differentiation protocols [14]. While beyond the scope of this review, there is also an increasing awareness of producing regenerative cell therapies in vivo, with improved tolerability, as previously demonstrated by Li et al. [15] where HSCs were transduced in vivo using helper-dependent adenoviral vectors.

This review focusses on the recent advances of regenerative cell therapies using haematopoietic cells and the ex vivo cell culture methods that have enabled them.

## 2 Advances in the landscape

### 2.1 Regenerating the haematopoietic system

#### 2.1.1 Umbilical cord blood-haematopoietic stem cell transplantation

Haematopoietic rescue of lethally irradiated mice following syngeneic bone marrow grafts demonstrated that a rare bone marrow cell population possessed the unique capacity for both multipotent differentiation and self-renewal [16]. Later, the ability to regenerate the haematopoietic system was discovered with the observation of the colony forming unit (CFU) in the spleen of murine bone marrow transplant recipients [17]. A rare population of cells were responsible for the formation of donor-derived cell colonies consisting of multiple haematopoietic lineages as well as cells that retained parental colony-forming abilities across mitotic divisions. This led to the concept of the bona fide HSC, defined functionally by a multipotent nature allowing for long-term multilineage reconstitution of the entire haematopoietic system as well as theoretically unlimited self-renewal capabilities facilitated by in vivo engraftment potential. The specific identification of haematopoietic stem and progenitor cells (HSPCs) was enabled by the advent of multi-colour flow cytometry, to assess specific cell-surface marker expression such as the lineage-CD34^+^CD38^−^CD90^+^ fraction with the later addition of CD201+ amongst other markers [18]. Together this suggested a possible therapeutic strategy of transplanting the regenerative stem cell source, the HSC, to reconstitute the haematopoietic system, theoretically over the lifetime of an organism.

Since the first demonstration of myeloablative conditioning and allogeneic HSCT using matched donors as a curative treatment for leukaemia [4], HSCT and reconstitution of a healthy haematopoietic system has become standard clinical practice in the treatment of haematological malignancy. Therapeutic potential also includes the induction of allograft tolerance after organ transplantation [19] or the correction of heritable haematological disease with gene-editing such as sickle cell disease [20]. Sources of HSCs for transplantation beyond bone marrow have been explored, including umbilical cord blood (UCB). These HSCs exhibit advantages such as higher proliferative capacity and decreased incidence of graft versus host disease (GvHD) in patients receiving umbilical cord blood-haematopoietic stem cells (UCB–HSCs) compared with bone-marrow derived HSCs [21]. This has the potential to increase donor accessibility due to less rigorous requirements for human leukocyte antigen (HLA) matching. Despite this, obtaining the required therapeutic cell dose to achieve therapeutic engraftment remains a challenge due to the lower cell quantity from UCB.

To address these limitations, recent cell culture protocols have focussed on the selective ex vivo expansion of CD34^+^ UCB-HSCs with investigation into retention of functionality including bone marrow homing, long-term in vivo engraftment, self-renewal and multipotent haematopoietic reconstitution capacity. This is particularly important as current cell-surface marker identification is unable to definitively discriminate between HSCs that retain these characteristics and progenitor cells which function in the shorter-term and thus cannot regenerate the haematopoietic system for the lifetime of the patient. Therapeutically promising culture systems have involved medium supplementation with small molecule modulators such as nicotinamide (NAM) [22] and UM171 [9]. These molecules likely function through multiple mechanisms such as decreasing reactive oxygen species (ROS) concentration or improving mitochondrial metabolism to promote stemness. This was demonstrated by increased transcript levels of sirtuin-1 and HIF1-a, accompanied by decreased ROS concentrations in HSCs cultured at low NAM concentration. These genes are implicated in the hypoxic stress response which arises during ex vivo culture due to proliferation and expansion. It has also been postulated that they promote HSC stemness, inhibiting differentiation, through altering gene expression such as increasing the expression of stemness-related genes such as BMI1, a component of polycomb repressive complex-1 or HOXB4 transcription factor [23]. UM171 has been suggested to enhance self-renewal and lineage skewing later at the erythroid-megakaryocyte-mast progenitor (EMMPs) stage towards a mast cell fate through inducing a mast-cell specific transcriptional programme in EMMPs [24]. This transcriptional change may be due to regulation of histone modifications where UM171 has been demonstrated to mediate the proteasomal degradation of the CoREST histone deacetylase complex via polyubiquitination of the lysine-specific histone demethylase-1 (LSD1) and REST corepressor 1 subunits to retain H3K4 methylation patterns at genes indicative of an undifferentiated HSC state [25]. A recent cryo-electron microscopy structure of the LSD1-histone deacetylase 1(HDAC1)-CoREST complex with UM171 and KBTBD4 homodimer, the substrate adaptor for the CUL3-RING E3 ubiquitin ligase, demonstrates UM171 stabilising the interaction between HDAC1 and KBTBD4 through binding into a complementary pocket formed at the interface of HDAC1 and a KBTBD4 protomer to facilitate polyubiquitination and subsequent proteasomal degradation [26]. Cui et al. have also reported enhanced expansion of phenotypically defined HSPCs and short-term HSCs (ST-HSCs) as well as synergistic effects on long-term HSCs (LT-HSCs) when cultured in the presence of both NAM and UM171. Maintenance of self-renewal and multilineage differentiation was demonstrated in colony forming cell assays (Y. [27]), suggesting the dependence on multiple pathways for robust HSC expansion.

In addition to small molecule supplements of culture medium, polymer-based culture systems have been pivotal in enabling long-term ex vivo expansion of human UCB-HSCs to produce a therapeutically relevant cell dose for HSCT as well as a platform for fundamental research into HSC characteristics or the application of gene-editing protocols for corrective therapies. The use of caprolactam polymer ‘Soluplus’ allowed for the remarkable 55-fold selective long-term expansion of CD34^+^ human UCB-HSCs achieved after a 30-day culture in a chemically defined ‘3a-medium’ as compared with the 10-fold selective expansion achieved by a standard polyvinyl alcohol-based polymeric culture system. These CD34^+^ HSCs retained engraftment potential upon murine xenotransplantation, self-renewal and multipotent differentiation capabilities [9]. A further development of this system included the addition of bortezomib and tumour necrosis factor-related apoptosis-inducing ligand with lenalidomaide for the expansion of both UCB-HSCs and peripheral blood-mobilised HSCs while completely eradicating contaminating multiple myeloma cells within the HSC graft, introducing further therapeutic alternatives for autologous HSCT in the treatment of multiple myeloma [28].

An example of an ex vivo expanded cord blood regenerative therapy with NAM-supplemented culture is omidubicel [29]. Clinical trials demonstrated a median 130-fold expansion of CD34^+^ cells in the omidubicel-treated grafts accompanied by a significantly faster neutrophil engraftment and platelet recovery reflected in a lower rate of infection compared with control. This culminated in the 2023 Food and Drug Administration approval of omidubicel for patients with haematological malignancies requiring an UCB-HSCT. Advances in maintaining cell viability and ex vivo expansion of UCB-HSCs have immediate implications in transplantation therapies for increasing donor availability for patients by enabling greater tolerance of HLA antigen mismatches as well as a single UCB donor that is sufficient for multiple recipients, streamlining the HSCT treatment landscape.

#### 2.1.2 Genome modified and gene-edited HSCs

Genome modification and gene editing enables autologous HSCT through the ex vivo correction of disease-causing mutations in patient HSCs. This approach to regenerating a haematopoietic system doesn't rely on donor availability and mitigates GvHD risk due to complete matching of HLA. Gene therapies for autologous HSCs with monogenic mutations tend to rely on integrating retrovirus or clustered regularly interspaced short palindromic repeats/cas9 (CRISPR/cas9)-based gene editing systems. In conditions caused by loss of function this will entail wildtype gene introduction while gain of function or dominant negative conditions may require knock-out of the mutant gene [30]. Examples include, Strimvelis, the first stem-cell based gene therapy, an autologous CD34^+^ HSC product with wildtype adenosine deaminase (ADA) sequence introduced by a ɣ-retroviral vector for the treatment of ADA-severe combined immunodeficiency [31]. This was followed by the first approval of a CRISPR/cas9 HSC therapy, Casgevy, in the treatment of sickle cell disease and transfusion-dependent thalassemia, by targeted disruption of the enhancer regulating BCL11A, a repressor of foetal haemoglobin (Hb) thus inducing functional foetal Hb expression [32]. However, several barriers have limited the widespread adoption of HSC gene therapies. These include inefficient viral transduction [10] and gene-editing [33] as well as the random integration of transgenes [34] or off-target gene-editing effects [33] which contribute to cellular heterogeneity and may pose oncogenic risks.

Similarly to HSC expansion protocols, organic compounds have been trialled during ex vivo culture either to increase transduction efficiency, so-called ‘transduction enhancers’ or to modulate HSC properties. Notably, Jang et al. investigated poloxamer F108 (LentiBoost), prostaglandin E2 (PGE2), LentiBoost + PGE2 and cyclosporine H during lentiviral transduction [10]. Although all four conditions enhanced vector copy number (VCN) as measured in culture, following murine xenotransplantation, only LentiBoost and cyclosporine H produced engraftable, functional HSCs with significantly increased VCNs. Later reports have suggested that epigenetic regulators; quisinostat, CPI203 [35] and UM171 [36] have not only enhanced transduction efficiency but also promoted HSC expansion and regulated downstream lineage output. For example, HSCs cultured with CPI203 exhibited lymphoid lineage priming as observed by increased frequencies of donor-derived common lymphoid progenitors, pro-B cells and CD19^+^ B cells in the bone marrow and CD3^+^ T cells in the thymus of transplanted mice, whereas UM171 with stem cell factor (SCF) and vascular endothelial growth factor (VEGF) specifically expanded a CD31^+^CD14^+^ monocyte population compared with established protocols. Interestingly, Tajer et al. demonstrated that while lentiviral transduction alone induces HSC proliferation, addition of small molecules such as quisinostat or CPI203 modulate HSC function by promoting maintenance.

In addition to viral transduction protocols, attempts to improve editing efficiency in HSPCs have been investigated with the use of cell intrinsic modifications. Barcode introduction and clonal tracking by BAR-seq elucidated cell intrinsic modifications including over-expression of adenoviral proteins and transient p53 inhibition using a dominant negative mutation to improve editing efficiency and maintain clonality of the edited HSPCs respectively while maintaining long-term self-renewal and multi-lineage reconstitution. Furthermore, the addition of small molecule cell culture modulators such as StemReginin 1 and UM171 for the extended culture of UCB-HSPCs post-editing demonstrated an increased proportion of phenotypic HSCs at the end of the culture period as well as improved human engraftment upon murine xenotransplantation. This approach suggests an additional consideration of HSPC heterogeneity and arising clonal dynamics upon editing where reduced clonality in HSCT is associated with slower haematopoietic reconstitution and decreased graft safety [37]. Protocols that improve genome modification or gene editing efficiency hold promise for regenerative therapies by generating HSC grafts that consist significantly or wholly of corrected HSCs, providing a solution when a threshold proportion of corrected cells are required for therapeutic effect such as sickle cell disease [38]. This may also enable the applicability of corrected HSCs in the treatment for pathology of polygenic origin where multiple gene edits are required. Combining these protocols with the future potential for single human HSC screening and culture could ensure the selection of desired, modified or edited cells while avoiding cells with deleterious off-target effects from genome modifying or editing procedures, affecting cell function.

Ongoing clinical trials are continuing to explore gene therapies in the context of autologous HSCT. This includes for the treatment of transfusion-dependent thalassemia through lentiviral insertion of a construct named ALS20 consisting of the beta-globin coding sequence with a T87Q mutation, a shortened promoter and a locus control region with four hypersensitivity sites to achieve sufficient transgenic expression levels [39]. Alternative CRISPR-based technologies that offer greater specificity and precision are also being investigated such as prime editing in the treatment of autosomal recessive chronic granulomatous disease to correct a two nucleotide deletion in the NCF1 gene encoding p47phox protein, as demonstrated by 87 % of CD34^+^ LT-HSCs with at least a single edited allele sixteen weeks post-murine transplantation [40].

#### 2.1.3 ESC derived HSC

Despite the relative ease of sourcing HSCs, allogeneic HSCT presents problems with graft rejection [41], GvHD risks [42] and concerns over suitable donor supply [43]. Autologous HSCT can be costly and time-consuming to manufacture due to their relative resistance to genome modification or editing as well as their rarity, where CD34^+^ HSCs in bone marrow typically accounts for just 1 % of the total bone marrow cells [44], exacerbated in patients subject to previous myeloablative treatments. To overcome these limitations, human pluripotent stem cells, including ESCs and iPSCs, have been explored as alternative sources to derive HSCs due to their high proliferative capacity in culture.

Differentiation of hPSCs into HSCs has proven challenging with efforts aiming to recapitulate the events during early development where HSCs arise from the haemogenic endothelium (HE) situated in the aorta-gonads-and mesonephros (AGM) region, a key step in haematopoietic commitment [45]. Historically, induction of haematopoietic lineage commitment from ESCs involved serum-containing co-culture systems with murine bone marrow or human yolk sac endothelial cell lines [46] or the formation of embryoid bodies from ESCs followed by mesoderm specification with an array of cytokines such as bone morphogenic protein-4 (BMP-4) [47]. The usage of serum and feeder cells can preclude the clinical applications of these derived HSCs. Notably, Demirci et al., reported a serum- and feeder-free protocol for embryoid body-derived HSCs where single ESCs were cultured with BMP-4, basic fibroblast growth factor (bFGF), and later with SCF and VEGF [48]. Despite serum- and feeder-free protocols, these approaches have been unable to produce long-term, definitively engraftable multilineage HSCs.

Current efforts have investigated HSC production in gastruloid culture systems, 3-dimensional (3D) ESC aggregates that, unlike embryoid bodies, retain temporal and spatial organisation of the three germ layers and exhibit defined tissue patterning reminiscent of the developing embryo. Rossi et al. report the production of haematopoietic precursors from murine ESCs (mESCs) cultured in a defined gastruloid culture system with pulsing of a glycogen synthase kinase-3 inhibitor followed by culture with VEGF-, ascorbic acid phosphate- and bFGF- supplemented medium post-mESC aggregation. Consistent with developmental events, a population of c-kit+/CD34+ cells that later express CD41, a key developmental marker of early haematopoiesis, and a population of c-kit+/CD34+/Ter119+/CD41-erythroid progenitor cells were identified. In vivo repopulating capacity was demonstrated by transplantation of the gastruloid cells and subsequent formation of CFU in the murine spleen [49]. Together this demonstrates the importance of tissue patterning and the maintenance of temporal and spatial regulation to initiate the physiological biochemical signal gradients taking place during development for the faithful differentiation of HSCs from hPSCs.

Additional studies to understand the molecular level signalling events that drive HE specification and commitment to HSCs over HSPCs have further highlighted the importance of temporal and spatial regulation during HSC differentiation. A comprehensive culture system detailing the differentiation of hPSCs to HSPCs with high purity was reported by Fowler et al., following the developmental trajectory of posterior primitive streak development and mesoderm specification, giving rise to the HE. This is enabled by defined temporal regulation of developmental signalling pathways; BMP, fibroblast growth factor, retinoic acid, Notch, transforming growth factor-β and Wnt. Interestingly, this gave rise to HLF + HOXA5-10+ cells, markers of physiological HSCs, yet these cells experienced sub-optimal engraftment upon transplantation. It may be speculated that this is due to quantitative Notch signalling effects where Fowler et al. report Notch activation for HSC development [50] whereas other reports specify the requirement of weak Notch signalling activation [51].

#### 2.1.4 iPSC derived HSC

Building on these findings, researchers have turned to iPSCs as an alternative source for HSC derivation given their ethical advantages. Reprogramming adult somatic cells promises the production of unlimited quantities of allogeneic HSCs from patient-derived iPSCs without risk of rejection or GvHD as well as corrective therapies due to the amenable nature of iPSCs for gene editing [52]. Despite their non-physiological nature, iPSC-based HSC differentiation in ex vivo systems primarily seek to replicate HSC development in the AGM. Despite low initial yields of HSPC production from iPSCs with early differentiation protocols, investigation of cell surface markers and key transcription factors such as BRACHYURY and GATA2 [53], have confirmed mesoderm specification and developmental haematopoiesis with intermediate HE development for HSC differentiation from iPSCs [54]. Similar to ESC-derived HSC differentiation, iPSC-derived HSC differentiation has relied on co-culture with stromal cells [55] or embryoid body formation with cytokine supplementation [56].

The development of serum-free and feeder-free culture conditions have been important in the transition to chemically defined culture systems to improve experimental reproducibility and clinical application. These chemically defined conditions also enable the unravelling of key molecular components that underpin iPSC-derived HSC differentiation. A comparative approach between serum-free, feeder-free culture methods in 2-dimensional (2D) and 3D culture was investigated [57]. A 2D culture system published by Smith et al. produced high yields of CD45^+^CD34^+^ cells and highlighted the importance of prolonged Wnt with aryl hydrocarbon receptor signalling [58]. A more recent transgene-free and stroma-free protocol used a predictive computational model to quantitatively define combinations of supplementing growth factors; SCF, thrombopoietin (TPO), FLT3, BMP4, VEGF, Il-3, Il-6, Il-1β, granulocyte-colony stimulating factor and insulin growth factor-1 during culture of several iPSC cell lines culminating in transplantation of embryoid body cells. Retention of functional HSC characteristics could be observed by detectable engraftment after primary and secondary transplantations demonstrating self-renewal and multilineage differentiation capacity where human myeloid, B-cell, T-cell and erythroid precursor cells were detected in the recipient bone marrow [59].

Molecular characterisation of haematopoietic differentiation from iPSCs has also indicated specific chemical compounds necessary for regenerative therapy manufacture. A recent study focussed on the regulated involvement of multiple pathways during mesodermal specification and HE formation for HSC differentiation. Monolayers of iPSCs were cultured in basal medium supplemented with cytokines and additional pharmacological signalling modulators; Wnt activator (CHIR99021), Activin/Nodal pathway inhibitor (SB431542) and phosphatidylinositol 3-kinase inhibitor for downstream mitogen-activated protein kinase activation (LY294002) at defined timepoints. Cells treated with all three pharmacological signalling modulators showed synergistic effects on HE formation which are within the CD43^−^CD45^−^cell population accompanied by significantly increased CFU forming abilities of produced CD43^+^CD34^+^ HSPCs in primary and secondary clonogenic assays, however long-term engraftment following transplantation was impaired [60]. The importance of temporal control for developmental signals has also been a concern in 3D cultures where varying sizes of embryoid bodies leads to variation in growth factor diffusion rates which may alter differentiation trajectories. Bello et al. explored the stepwise action of growth factors for mesoderm induction followed by haematopoietic specification during iPSC differentiation into HSCs, using fast and slow-release gelatin microparticles to control the sequential release of BMP4 and SCF. Cultures with growth factors released by gelatin microparticles had higher expression of mRNA and proteins associated with mesodermal and haematopoietic lineages such as CD34 and CD133 [61].

#### 2.1.5 Challenges of hPSC-derived HSCs

The faithful differentiation of HSCs from hPSCs is a key obstacle in haematopoietic regenerative therapy. The potential establishment of allogeneic hPSC-derived HSC banks could enhance flexibility and accessibility for HSCT. Yet, the reliable production of HSCs with long-term in vivo engraftment, self-renewal and multilineage reconstitution capabilities, LT-HSCs, remain challenging, exacerbated by the heterogenous nature of differentiation observed in current culture systems. For HSCT success in the clinic there is a requirement for rapid in vivo engraftment and reconstitution which can be mediated by progenitor cells and ST-HSCs for the fast recovery of haematopoietic cells, such as initial neutrophil and other myeloid cell recovery [62], to prevent infection, a leading cause of mortality post-HSCT [63]. It is also important to ensure the simultaneous transplant of LT-HSCs that will enable long-term HSCT success through continuous self-renewal and multilineage reconstitution for the sustained regeneration of a healthy haematopoietic system including erythrocytes, megakaryocytes, myeloid and lymphoid lineage immune cells. As the detection of cell-surface marker expression alone is insufficient in determining LT-HSCs, this necessitates robust functional and molecular assays in the research context to evaluate the suitability of hPSC-HSC for regenerative therapies.

Current benchmarks for defining bona fide HSCs include in vivo assays such as single-cell murine xenotransplantations of fluorescence activated cell sorting purified single cells to demonstrate long-term engraftment and sustained multilineage reconstitution over a period of at least 16 weeks [64]. Commonly long-term engraftment, self-renewal and multilineage reconstitution are assessed in serial murine xenotransplantations whereby the presence of phenotypic HSCs in the bone marrow and secondary lymphoid organs as well as donor-derived lineage output in the bone marrow, secondary lymphoid organs and peripheral blood are detected at least 16 weeks following secondary or tertiary transplantations [65]. As molecular investigation at single-cell resolution continues to advance, it is possible to correlate a molecular to functional state of an HSC [66] including single cell RNA sequencing (scRNA-seq) or multimodal analysis such as the integration of cell-surface marker expression with transcriptomic data at a single cell level by cellular indexing of transcriptomes and epitopes by sequencing [67]. Bioinformatic methods that use molecular states to predict functional activity and thus HSC determination may be particularly helpful in the development of regenerative therapies through the screening of clones expanded from single hPSC-HSC. However, limitations to these functional assays may also limit inferences that can be made from a laboratory to a clinical setting including the use of immunocompromised murine models for transplantation such as a non-human HSC niche which may affect HSC function and lineage output as well as molecular assays only accounting for HSC state at a singular point in time as opposed to function over time.

### 2.2 Regenerative therapies generating a specific haematopoietic cell type

Regenerative therapies can generate specific differentiated haematopoietic cell types from stem cells as well as restoring the entire haematopoietic system. This may aim to replace a lost function of a specific haematopoietic cell type such as in the management of immunodeficiency [68], anaemia [69] or thrombocytopenia [70]. Alternatively, this may provide an enhanced [71] or novel cellular function [72] to improve clinical applicability and enable the therapeutic mechanism of action. Regenerative approaches enable large-scale production of therapeutic cells with a naïve phenotype, difficult to achieve for terminally differentiated haematopoietic cells from peripheral blood [73]. In vitro cell differentiation protocols for specific haematopoietic cell types have been developed that focus on scalability, clinical applicability regarding safety, GMP-compliance and regeneration of physiological functionality.

#### 2.2.1 T cells

**Fig. 1:**
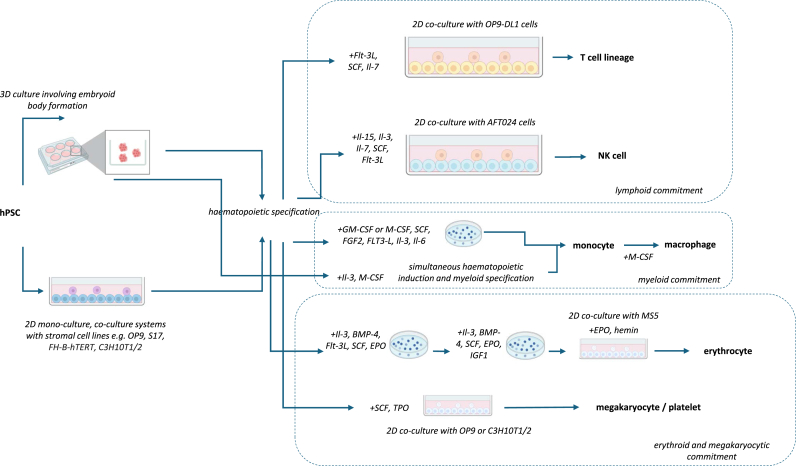
Principles behind classical ex vivo differentiation culture systems.

Viral or tumour antigen-specific T cell transfer [74] are being explored for use in the treatment of immunodeficiency, viral infection or cancer. Stem-cell derived T cells, unlike mature peripheral T cells, exhibit a naïve phenotype characterised by self-renewal capacity and rapid differentiation into effector subsets [71]. This is a particular challenge for the ex vivo culture of primary T cells where culture systems often promote their terminal differentiation and exhaustion prior to clinical application [75], limiting their effector functionality. Initial in vitro T cell differentiation cultures followed a two-step method from hPSCs through co-culture with murine stromal cells OP9 for haematopoietic commitment followed by co-culture with OP9-DL1 cells to induce Notch signalling to specify T cell differentiation [76] (Fig. 1). A regenerative approach is also advantageous in the production of antigen-specific T cells due to the amenability of hPSCs to genome modification and gene editing protocols as well as rapid expansion during differentiation for clinically relevant T cell supply. However, there have been challenges in efficiently differentiating ⍺β-T cells over innate-like ɣδ-T or invariant natural killer T cells that are unable to recognise HLA-restricted antigenic peptides [23,77].

Recent research efforts have focussed on identifying the minimum necessary molecular components to achieve a chemically defined system to improve reproducibility and scalability of regenerative therapy manufacturing. Examples have included the use of DLL4 coated plates [78] and suspension of DL4 conjugated microbeads in culture medium [79]. Building on previous differentiation models, Ito et al. derived iPSCs from tumour infiltrating lymphocytes (TILs) isolated from patient tumour samples followed by in vitro differentiation into T cells. Embryoid body formation followed by feeder-free culture on Fc-DLL4 coated plates gave rise to double positive (CD4^+^CD8^+^) thymocytes that, with additional CD3 stimulation, led to single positive T cell differentiation. The produced CD8⍺β T cells retained the same antigen specificity as the TILs and expressed subset-specific markers such as CD62L for central memory T cells, associated with improved anti-tumour efficacy [80] and natural killer (NK) cell-related marker expression like CD336 and CD337, also implicated in cytotoxicity. Functional investigation when co-cultured with autologous, patient-derived cancer spheroids revealed TIL-iPSC-T cell cytotoxicity against the cancer spheroids was not ablated when inhibiting HLA-Class I molecule interactions, suggesting innate cytotoxic functionality, possibly mediated by the expressed NK cell-related markers [78]. Iriguchi et al. also used a similar two-step protocol in the differentiation of T cells from iPSCs but due to low efficiency of double positive T cell formation, used additional medium supplements of CXCL12 and a p38 inhibitor. This demonstrated a synergistic effect, inhibiting apoptosis of developing T cells and improving the differentiation efficiency of double positive and immature single positive cells. This culture method was also demonstrated to be compatible with lentiviral transduced iPSCs with T cell receptor (TCR) ⍺ and β chains specific to an HLA-A∗24:02 restricted Wilms tumour-1 specific peptide, with 75 % of CD4^−^CD8⍺β+ cells maintaining TCR specificity [72]. Expansion of these iPSC-derived T cells also successfully took place under serum-free conditions while faithfully retaining expression of CD4 and CD8⍺.

Gene-editing approaches have been explored to further understand the genes involved in faithful T cell differentiation and inform cell-intrinsic modifications that can be made to improve differentiation efficiency or enhance therapeutic function. CRISPRi-mediated knockdown of enhancer of zeste 1 polycomb repressive complex 2 subunit (EZH1) methyltransferase, in iPSC-derived CD34^+^ HE cells during stroma-free T cell specification increased T cell yield and CD3+TCR⍺β+ cells over CD3+TCRɣδ+ cells both by immunophenotypic and transcriptional analysis [81]. These EZH1 knockdown iPSC-derived T cells most closely resemble peripheral blood ⍺βT cells in terms of their transcriptional signature and, specifically upon T cell stimulation, exhibit a memory-like T cell phenotype as revealed by scRNA-seq. This suggests that epigenetic reprogramming within a specific developmental window can broadly improve the efficiency of CD3+TCR⍺β+ T cell differentiation as well as regulate T cell differentiation into specific subsets in response to antigen stimulation, with implications for the sustained efficacy of adoptive stem-cell derived treatments.

Despite the challenges in achieving specific T cell differentiation, advancements in gene editing and culture systems have enabled the production of T cells with enhanced functionality, making them a promising candidate for immunotherapy applications.

#### 2.2.2 NK cells

NK cells have recently garnered attention as immunotherapies. They have improved safety over T cells including their lower risk of cytokine release syndrome, neurological toxicities and GvHD due to their HLA-independent mechanism of cytotoxicity, allowing for their allogeneic use as an ‘off the shelf’ therapy [82]. Like T cells, stem-cell derived NK cells can produce large cell quantities and enables a platform for the incorporation of genetic modification and gene editing protocols during therapy manufacture. In 2005, a two-step co-culture method generated NK cells from ESCs through haematopoietic induction by co-culture with firstly the S17 followed by AFT024 stromal cell lines in medium supplemented with cytokines including Il-7 and Il-15 (Fig. 1). These cells expressed several killing inhibitory receptors (KIRs) and a killing activating receptor (KAR) suggesting NK lineage differentiation [83]. Other protocols for differentiation from ESCs and iPSCs have explored feeder-free embryoid body formation to obtain HE cells followed by lymphoid specification [84]. While regenerative NK cell therapies offer advantages such as HLA-independent cytotoxicity, they are hindered by their limited in vivo persistence and challenging differentiation protocols [85]. To address this, recent efforts have focused on elucidating the molecular pathways that promote NK cell differentiation and viability for efficient and standardised differentiation of physiologically relevant NK cells displaying full effector functionality and to inform relevant genetic modifications of hPSCs to enhance in vivo persistence [86].

Euchner et al., reported a protocol for the robust and efficient generation of NK cells from iPSCs using an embryoid body protocol followed by co-culture with OP9-DL1 cells, producing CD45^+^ cells with 94 % purity by week three of co-culture. Phenotypic and in vitro characterisation further revealed expression of phenotypic NK marker CD56, activation marker CD69 and maturation marker DNAM-1 but the absence of a second maturation marker, CXCR1, suggests impaired maturation into canonical CD56^dim^CD16+ NK cells by this culture system. Interestingly, the KIRs, KIR2DL2/L3 and KIR3DL1, were detected in a minor cell population despite notoriously challenging KIR expression in ex vivo differentiation cultures due to the requirement of HLA interaction for education which may have been mediated by a stromal cell interaction in this case. The KAR, NKG2D, and natural cytotoxicity receptors, NKp46, NKp44 and NKp30 were also expressed, accompanied by functional cytotoxic activity [87].

To uncover the molecular pathways taking place during NK cell differentiation and survival, a scRNA-seq and computational approach to unravel the gene regulatory networks between in vivo differentiated NK cells from the foetal liver and iPSC-NK cells was taken. In-depth transcriptomic analysis revealed STAT5A as a key regulator of NK cell differentiation [88]. The involvement of STAT5 for NK cell viability could also be seen in an investigation of cell-intrinsic characteristics, by a CRISPR/cas9 mediated knock-out of the cytokine-inducible SH2-containing protein (CISH) gene, responsible for antagonising Il-15-JAK-STAT signalling in iPSCs. CISH knockout iPSC-NK cells displayed enhanced survival in vivo as well as increased STAT5 phosphorylation levels in response to in vitro activation with Il-15 as compared with control iPSC-NK cells [89]. These NK cells were differentiated ex vivo from iPSCs using an embryoid body protocol as described by Zhu and Kaufman, [84], highlighting the importance of incorporating gene-editing techniques to established NK cell differentiation cell culture pipelines to facilitate the development of more efficacious cell therapies through either the direct manipulation of cell characteristics or to elucidate signalling pathways that can be exogenously manipulated during therapy manufacture.

In addition to molecular components, biophysical effects have also been demonstrated to enhance NK cell differentiation. Wen et al., utilised a two-step culture system; UCB-HSC expansion followed by co-culture with OP9-DLL1-DLL4 cells for NK cell differentiation while supplementing the medium with an Na + -containing solution to demonstrate the effects of osmotic pressure on NK cell differentiation efficiency. An increased osmotic pressure, of 345 mM and 360 mM, increased both the NK cell output per UCB-HSPC and proportion of CD3^−^CD56^+^ cells in culture respectively. The UCB-HSC derived NK cells significantly decreased expression of inhibitory receptors such as CD94 and significantly increased expression of activation markers such as CD69. Transcriptomic analysis of the NK cells differentiated under osmotic pressures of 330 mM revealed few differentially regulated genes beyond NK cell activation and cytokine responses compared with NK cells cultured under standard osmotic pressure suggesting that osmotic conditions primarily affect the efficiency of NK cell production and their activation-induced functionality [90].

NK cells are key players in innate immunity and are increasingly being explored as adoptive cell therapies. Stem cell-derived NK cells are an attractive candidate for a regenerative cell therapy due to their cytotoxic and immune modulating capabilities with an improved safety profile over T cell-based therapies.

#### 2.2.3 Monocytes and macrophages

While NK cells offer significant promise, functions of other innate immune cells also allow for their suitability as adoptive cell therapies. Macrophages, a myeloid lineage immune cell, have been reported to be highly reactive to their environment through sensing of environmental factors and altering their phenotype in response to promote tissue homeostasis. This may be via the recognition and phagocytosis of aberrant cells such as in cancer immunotherapy or facilitating tissue regeneration through the secretion of anti-fibrotic or anti-inflammatory molecules into the extracellular environment such as in liver fibrosis. Macrophage subsets include M1 involved in pro-inflammatory responses and M2 involved in anti-inflammatory responses [91]. Transfer of regenerated macrophages is beneficial due to their ability to be produced in large quantities as well as having been reported to exhibit naïve-like cytokine secretion profiles enabling a robust pro-inflammatory response to pathogens or tissue damage [92]. Like other haematopoietic cells that have undergone ex vivo differentiation from hPSCs, macrophage differentiation culture depends on the premise of mesoderm induction, HE specification with simultaneous myeloid lineage commitment resulting in the formation of CD14^+^ monocytes and subsequent terminal differentiation into macrophages. Early systems relied on either 2D co-culture systems with stromal cells such as OP9 cells or on 3D culture systems for embryoid body formation with myeloid commitment promoted by IL-3 and macrophage-colony stimulating factor (M-CSF) medium supplementation [93] (Fig. 1).

Serum-free and feeder-free conditions for the ex vivo differentiation of haematopoietic monocyte-derived macrophages have been relatively well-established [94] and recent developments of cell culture methods aim to develop scalable production of stem-cell derived macrophages with high efficiency. An example by Ackermann et al., is the investigation of the scalable production of iPSC-derived macrophages using a 3D culture system. iPSC colonies expanded in 2D culture were separated and cultured in 3D small-scale or large-scale culture in stirred-tank bioreactors with Il-3 and M-CSF supplemented medium. Produced cells displayed a CD45^+^CD11b^+^CD14^+^CD163^+^TRA-1-60^-^ cell-surface marker phenotype by flow cytometric analysis while an in vitro functional assay suggested their ability to phagocytose fluorescent bioparticles [95].

A challenge is ensuring macrophages retain a therapeutic phenotype and not the adoption of a phenotype advantageous for advancing pathology, a concern due to their highly responsive and plastic nature. A recent study compared a 2D serum-free, mouse embryonic fibroblast feeder cell culture system with feeder cells subsequently depleted to a 3D embryoid body culture system for the ex vivo differentiation of macrophages from iPSCs. RNA-sequencing was used to assess whether macrophage phenotype is dependent on ex vivo differentiation method. While iPSC-macrophages derived by both methods demonstrated co-expression of M1 and M2 markers, notably, macrophages differentiated by 2D culture showed significant upregulation of genes involved in anti-inflammatory responses associated with M2 macrophages whereas macrophages differentiated by embryoid bodies tended to show upregulation of pro-inflammatory associated genes. This was consistent across two different iPSC lines [92]. This suggests that, depending on the therapeutic application and the desired macrophage phenotype, a specific differentiation culture method may be preferable with potential for regenerative therapy manufacture through large-scale culture.

#### 2.2.4 Erythrocytes

Erythrocytes are the most transfused cell type for example in the treatment of genetic Hb pathologies such as sickle cell disease, acquired anaemias in haematological malignancy or as adjuncts to surgery [96] necessitating scalable production strategies. Erythrocytes directly from stem cell sources from a limited number of stem cell donors separated by blood type, as opposed to relying solely on primary blood donations, would offer a solution for a theoretically unlimited supply of cells, even for rare blood types. In addition, these regenerated erythrocytes may have enhanced function such as survival, investigated in a Phase I clinical trial [97] for ex vivo regenerated erythrocytes. In 2002, the first serum-free culture system giving rise to erythroid precursor cells from CB-derived CD34^+^ cells through serum-free expansion of CD34^+^, erythroid progenitor cells and erythroid differentiation was reported [98]. Erythroid cell differentiation from hPSCs aimed to recapitulate developmental erythropoiesis either using 2D mono-culture, co-culture or 3D embryoid body dependent systems. Olivier et al. differentiated embryonic erythroid cells through the co-culture of ESCs with a human foetal liver cell line for haematopoietic specification followed by three successive steps for erythroid differentiation and maturation in medium supplemented with cytokines such as SCF, BMP4 and erythropoietin (EPO) [99]. Despite this progress of delineating the required signals for erythropoiesis ex vivo, obstacles remain in producing clinically relevant and functional erythrocytes such as faithfully recapitulating the hallmark events of erythropoiesis such as switch of Hb expression from foetal ɣ-Hb to stable adult β-Hb expression and high-yield enucleation.

Soares-Martins et al. conducted a comparative study looking at 2D or 3D culture protocols for the haematopoietic commitment step for erythroid production from donor-derived iPSCs and demonstrated efficient enucleation rates in vitro. The 2D culture used a serum-free system with medium supplemented with cytokines including bFGF, BMP4, VEGF and CHIR99021 and the 3D cultures utilised embryoid body formation with medium supplemented with bFGF, VEGF, SCF and BMP4. While the authors were not able to reproduce efficient HSPC induction from the previously reported 2D monolayer culture, the 3D cultures produced CD34^+^CD43^+^ cells at approximately 50 % purity by the end of the embryoid body formation culture period. There was variation between iPSC lines, suggesting that possible heterogeneity between iPSC lines may require specific protocols [100] for efficient differentiation of erythrocytes, thus suggesting the importance of validation and screening of iPSC lines used for regenerative therapy manufacture.

To elucidate the regulation underlying enucleation, Deng et al. utilised peripheral blood mononuclear cell-derived iPSCs which were in vitro differentiated into erythroblasts, followed by an eight-day culture period in medium supplemented with EPO and either human plasma lysate, human serum or plasma albumin. After the culture period, all three culture conditions exhibited enucleated erythroblasts at a proportion of approximately 13 %, yet, upon transfusion of NOD-scid-gamma immunodeficient mice with iPSC-erythrocytes, it could be seen that there was a rapid increase in enucleation rates up to >90 % a day post-transplant. This suggested the requirement of in vivo interactions, beyond serum factors, for high efficiency enucleation such as erythroblast-macrophage dependent and independent interactions [101]. Soares et al., demonstrated by flow cytometric analysis, following the final erythroid maturation stage in EPO-supplemented medium, that 95 % of CD235+ cells were negative for deep red anthroquinone-5, a nuclear stain, suggesting that efficient enucleation had taken place in this system. However, the switch from foetal to stable adult Hb expression was more challenging to achieve, with RT-qPCR analysis of total cells from the erythroid precursor differentiation stage revealing a foetal cellular phenotype with higher relative ɣ-Hb expression and lower relative β-Hb expression than adult peripheral blood erythrocytes [100].

Olivier et al., aimed to improve reproducibility as well as decrease costs during large-scale clinical manufacture of iPSC-derived RBCs. A chemically defined, cytokine-free culture system was developed through the gene editing of iPSCs to remove cytokine dependence of proliferation and erythroblast differentiation. CRISPR-cas9 was used to introduce kitD816V and jak2V671F mutations into iPSCs for constitutive signalling through the SCF receptor and JAK2. Gene-edited iPSC-HPSCs were then subject to erythroid differentiation with medium supplemented with small molecules dexamethasone, to promote self-renewal, and 3-isobutyl-1-methylxanthine to promote self-renewal and haematopoietic specification. Combined, these gene edits and ex vivo culture system enabled the differentiation of gene-edited iPSCs to self-renewing erythroblasts in a cytokine-free environment, specifically independently of SCF and EPO, for the rapid continued production of RBCs over 120 days, which could be reproduced across two different iPSC lines [102].

#### 2.2.5 Megakaryocytes and platelets

Megakaryocytes are terminally differentiated, HSC-derived polyploid cells formed by successive rounds of endomitosis. These precursor cells then mature by thrombopoiesis to produce platelets which have surveillance, haemostatic and inflammatory functions. Platelet transfusions are required for patients suffering from thrombocytopenia, a common complication from cancer treatment or severe blood loss [103]. Despite this demand, there are challenges to supply including their short storage life, donor shortages and, consequently, common antigen mismatching between donors and recipients, leading to rejection in some patients [104]. Deriving platelets from stem cells would allow for a theoretically unlimited supply of megakaryocytes and platelets with a defined antigenic profile allowing for stable turnover of stocks at allogeneic banks. After differentiation of hPSCs to HSPCs, as for other culture systems, megakaryopoiesis and thrombopoiesis from HSPCs involved SCF and TPO in co-culture with OP9 or C3H10T1/2 stromal cell lines to improve efficiency of differentiation [105]. These systems in combination with various cell-intrinsic modifications such as the introduction of a tetracycline inducible over-expression system of transcription factors GATA1, TAL1and FLI1 [106] by a non-viral method were able to achieve a higher yield of ex vivo platelets however inefficient thrombopoiesis, between one to two orders of magnitude lower than in vivo platelet production [105], in combination with the vast numbers required for transfusion, remain a problem [107].

Zhao et al. perfused cultured murine megakaryocytes into an ex vivo murine heart-lung system and was able to demonstrate comparable platelet production per megakaryocyte as in vivo thrombopoiesis, suggesting that the microenvironment including oxygen concentration may be important in this regulation at least for platelets produced in the lung vasculature. The produced platelets demonstrated phenotypic marker expression of CD61 and CD42b, albeit a reduced proportion expressing the latter marker as compared with control murine platelets, and characteristic spatial distribution of ⍺-tubulin and thrombus formation in vitro [108]. The importance of the microenvironment for platelet production has been applied to the design of large-scale cultures. An example is the development of a 3D baffle-flow and orbital rotator culture system for the eighteen-day culture of ESCs into mature megakaryocytes and platelets under chemically defined conditions to allow for increased and uniform oxygenation while improving reproducibility of the culture components. The baffle-flow conditions significantly increased the yield of phenotypic platelets CD41a + CD42b + cells per ESC, expression of megakaryopoiesis-related genes and mitochondrial membrane potential as compared with static 3D cultures [109].

#### 2.2.6 Safety barriers of iPSC-derived therapies

The use of hPSC-derived regenerative cell therapies also raises several safety concerns including cellular heterogeneity both within and between different iPSC lines spanning point mutations, larger chromosomal level mutations and abnormal epigenetic marks which may contribute to tumorigenesis, immunogenicity and cell rejection as well as aberrant haematopoietic differentiation [110]. To partially address these safety concerns, reprogramming approaches for iPSC generation in a clinical setting have focussed on non-integrating or non-viral approaches to ensure that reprogramming factors are expressed transiently, limited to the reprogramming window, as well as decreasing immunogenicity by ensuring the absence of viral proteins [111]. An increasingly popular approach is the introduction of reprogramming factors by episomal vectors and was demonstrated to be compatible with an oncogene-free system by Kamath et al. [112] where episomes become subsequently diluted over several rounds of cell cycle. Genetic instability during iPSC reprogramming, subsequent expansion and differentiation, particularly when oncogene c-myc has been used as a reprogramming factor, has been well-documented including point mutations and chromosomal instability. There may also be selective pressure within the iPSC line to accumulate mutations that promote cell proliferation so that mutant iPSCs rapidly become dominant clones in culture leading to concerns of tumorigenesis following transplantation [111]. Steps during manufacture to avoid the use of oncogenes during reprogramming such as c-myc or limiting the number of passages may also be used.

Due to these challenges, clinical application will require multiple quality control and safety checks, from the stage of donor somatic cell selection, iPSC colony selection, expansion and differentiation [113] both at a crude level through karyotyping and more sensitive methods such as whole genome sequencing or quantitative-polymerase chain reaction (qPCR) to detect the presence of specific tumorigenic mutations. Remaining pluripotent stem cells from incomplete differentiation pose further risks for tumorigenesis [114] through teratoma formation even at the order of magnitude of 1x10^4^ cells [111]. For regenerative therapy manufacture, the differentiation process can be assessed by flow cytometry or more sensitively by reverse transcriptase-qPCR (RT-qPCR) or digital PCR to assess the expression of hPSC specific gene expression such as OCT4 [115]. The current benchmark is the teratoma formation assay, utilising in vivo immunodeficient murine models to assess teratoma formation when iPSC-HSC grafts are transplanted. The incorporation of careful screening and rigorous safety checks during the manufacturing process contribute significantly to treatment costs, particularly in the case of autologous, patient-derived iPSC therapies. To counter this and to streamline manufacturing, iPSC banks consisting of screened, tested and GMP-grade iPSC stocks of known HLA expression to be used for regenerative therapy have been developed [110]. However, due to the genetic instability of iPSCs during expansion and differentiation culture it will also be important to frequently assess for the presence of tumorigenic mutations as well as highlighting the need to investigate whether culture conditions can modulate genetic stability.

## 3 Conclusions and future perspectives

In recent years, haematopoietic cells in the context of regenerative therapies marks a rapidly flourishing field with examples of first-in-class drugs entering the clinic as well as innovative therapy development undergoing pre-clinical investigation. Recent advances in ex vivo cell culture techniques have accelerated the field, allowing exploration of both allogeneic and autologous stem cell sources in combination with gene-editing protocols for the large-scale production of ‘off the shelf’ therapies as well as corrective approaches. Examples of cell culture techniques including serum-, feeder-free systems that are chemically defined with the addition of small molecule or polymeric supplements such as epigenetic or signalling pathway modulators, the use of 3D systems, or large-scale controlled bioreactor systems have aimed to establish standardised, reproducible protocols that are GMP-compliant. Compatibility of these protocols with gene-modifying or gene-editing protocols are becoming increasingly important for corrective approaches, to enhance cell function or to introduce novel therapeutic functions for their mechanism of action. Yet, despite the rapid progress of the field, key issues remain that hinder widespread clinical translation of pre-clinical studies.

A major limitation of current ex vivo differentiation approaches is the faithful recapitulation of in vivo differentiation to produce cells, either bona fide HSCs or differentiated cells, that retain physiological phenotype and functionality. The synergistic effects of medium supplements suggest that stem cell function and differentiation is regulated by a complex interplay of combinatorial factors in vivo which can be challenging to replicate in vitro. Heterogeneity inherent to the stem cells, genomic instability arising from iPSC generation and expansion or random insertion of transgenes during genome modification and off-target gene-editing effects can also lead to variable results for how ex vivo culture and differentiation may proceed that, affect cell functionality and lead to safety concerns of tumorigenicity or immunogenicity. This may also highlight an important opportunity for the development of predictive markers or models for the screening of individual stem cells at a genomic, transcriptional and proteomic level to assess which single cells may be selected for therapeutic development. Future research and direction to unravel these challenges to reproducibly and safely manufacture haematopoietic cell regenerative therapies will offer huge clinical promise across haematology, immunology and oncology.

Fig. 1 legend: Classical ex vivo directed differentiation protocols to produce lymphoid, myeloid and erythroid/megakaryocytic lineage cells from hPSCs rely on principles that govern developmental haematopoiesis. This involves initial mesoderm induction for haematopoietic commitment, giving rise to a primitive population of CD34^+^ cells. Typically, this involves either 3D culture systems for embryoid body formation or 2D mono-culture or co-culture systems with stromal cells. Following haematopoietic specification, specific signalling requirements, either provided by stromal cells in co-culture or as soluble medium supplements such as cytokines and growth factors, direct differentiation towards specific haematopoietic lineages. These early differentiation protocols have established a framework for the necessary steps and signalling requirements to achieve ex vivo differentiation for the application of recent cell culture, genome modification and gene-editing protocols.

Box 1 legend **Regenerative immunotherapies, using stem cells as a starting material, has provided ease and flexibility for the generation of CAR-based immunotherapies beyond CAR-T cells to achieve a variety of antigen-specific anti-tumour functionality due to their multipotency or pluripotency and amenability to genome modification or gene-editing. This has prompted exploration into the design of novel CARs for optimisation of function in different immune cell types. Following ex vivo differentiation, these stem0cell derived CAR immune cells can be adoptively transferred back into the patient.**

Regenerative immunotherapies, a growing branch of haematopoietic cell based regenerative medicine, is used in the treatment of various malignancies including leukaemias, lymphomas and more recently the exploration of solid tumours. The application of recent cell culture advances with gene modification protocols has enabled introduction of a variety of synthetic receptors [116], notably the chimeric antigen receptor (CAR), designed to target tumour specific antigens. Introduction of the DNA sequence encoding the synthetic CAR by viral transduction or CRISPR/cas9 gene-editing into stem cells followed by directed differentiation, traditionally to T cells, confers the T cells with antigen-specific but HLA-independent helper- or cytotoxic- T cell functionality, facilitating the anti-tumour mechanism of action. Regenerated (CAR)-T cells, like regenerated T cells, have additional benefits of exhibiting a stem-cell like, naive phenotype for robust effector functionality and can be used in an allogeneic context to counter challenges with sourcing primary T cells, particularly for patients subject to prior lymphodepleting therapies. For example, the ex vivo iPSC differentiation technique in serum- and feeder-free conditions demonstrated by Iriguchi et al. also produced iPSC-CAR-T cells expressing CD45RO and CD62L, suggesting a memory-like phenotype [72] while Harada et al. generated iPSC-T cells expressing both a CAR and an antigen-specific TCR demonstrating 100 % efficiency of CAR expression. This may be due to improved efficiency of viral transduction of iPSCs as compared with peripheral-blood T cells, reflected in significantly improved antigen-specific cytotoxicity against lymphoma cells [117].

CARs are also being explored in the context of other immune cells, enabled more easily by the pluripotent or multipotent nature of stem cells and relative susceptibility to genome modification or gene-editing. For example, iPSC-derived CD19-CAR-NK cells as demonstrated by Kong et al. target CD19-expressing brain pericytes, accessible in glioblastoma due to disruption of the blood-brain-barrier organisation, with lower risk of side-effects as compared with T cells [118]. Alternatively, CAR-macrophages have also gained interest due to their highly infiltrative and immunomodulatory nature. Shen et al., developed a 2D protocol for ex vivo differentiation of CD19-CAR-transduced hPSCs to CD19-CAR-macrophages with high efficiency of macrophage output per hPSC that exhibited stable CAR expression as confirmed by flow cytometry [119]. This has prompted novel CAR designs to improve intracellular signalling suitability for different immune cell types. This was previously investigated in NK cells through [120] demonstration of the superior antigen-specific cytotoxicity of an scFV-NKG2D-2B4-CD3ζ CAR as compared with a standard third generation CAR-T construct. This concept was applied to macrophages more recently by Shen et al. and Lei et al. where screening of CAR constructs has suggested that T cell specific co-stimulatory domains can hinder CAR functionality while CAR constructs with a CD3ζ-toll-like receptor 4 intracellular toll/Il-1R (TIR) showed enhanced and durable M1 polarisation to induce pro-inflammatory reprogramming of the tumour microenvironment [119,121].

Alt-text: Box 1 legend

## Declaration of generative AI and AI-assisted technologies in the writing process

During the preparation of this work the author(s) used ChatGPT in order to proofread. After using this tool/service, the author(s) reviewed and edited the content as needed and take(s) full responsibility for the content of the publication.

## Declaration of competing interest

All authors declare that they have no conflicts of interest.
